# The Transmembrane Glutamate Serves as a pH Sensor for Tha4 Oligomerization During Twin Arginine Transport of Proteins

**DOI:** 10.3390/plants14213338

**Published:** 2025-10-31

**Authors:** Vidusha S. Weesinghe, Christopher Paul New, Carole Dabney-Smith

**Affiliations:** 1 Department of Chemistry and Biochemistry, Miami University, Oxford, OH 45056, USA; 2 Cellular, Molecular, and Structural Biology Graduate Program, Miami University, Oxford, OH 45056, USA

**Keywords:** Twin Arginine Transport Pathway, Tha4, thylakoid membrane, proton motive force, voltage sensing, hydrophobicity, oligomerization

## Abstract

Tha4, the smallest component of the cpTAT system, is thought to be the pore-forming element in the TAT translocase. A conserved glutamate at the 10th position in its transmembrane helix is crucial for function. Substitution of this glutamate with alanine abolishes transport, while aspartate substitution partially restores it, highlighting the importance of charge and hydrophobicity. To examine these effects, we generated Tha4 variants with different glutamate substitutions and assessed their transport abilities. Additionally, we developed assays to evaluate Tha4 oligomerization in the presence or absence of a proton motive force (PMF) and functional precursor proteins. Glutamate positional substitutions designed to increase proximity to the acidified lumen were not tolerated in the alanine background, whereas aspartate variants showed slight tolerance. Oligomerization assays revealed that Tha4 oligomer formation in the transmembrane helix region was primarily dependent on the presence of a functional precursor, regardless of PMF, while C-tail oligomer formation responded mainly to PMF. The amphipathic region showed no significant response to either factor. Alanine substitution enhanced oligomerization, while aspartate reduced it, likely due to altered packing interactions between monomers. These discoveries highlight the crucial function of the transmembrane glutamate in sustaining Tha4 activity and ensuring appropriate assembly during activation transport.

## 1. Introduction

Chloroplasts are structurally intricate organelles composed of three membranes and three aqueous photosynthetic compartments, found in eukaryotic cells. The inner and outer envelope membranes of the chloroplast enclose the intermembrane space. The third membrane is the internal thylakoid, which is crucial to the organelle and is the site of photosynthesis. Within the chloroplast, around 3000 proteins are necessary for proper metabolic function, with approximately 97% of these proteins being nuclear encoded [1]. Among those proteins, more than 100 different thylakoid or thylakoid lumen proteins are encoded by genes in the nucleus and synthesized, acquiring spatial structure and functional activity in the cytoplasm as higher molecular weight precursors [2,3]. These proteins targeted to the thylakoid have a bipartite transit peptide that directs the protein first to the stroma, followed by the cleavage of the first signal sequence by a protease, which exposes the second signal sequence to direct the precursor protein further into the thylakoid membrane or lumen [4,5,6]. At the chloroplast’s outer and inner membranes, these newly synthesized precursor proteins first encounter the TOC (translocon on the outer chloroplast membrane) and TIC (translocon on the inner chloroplast membrane) translocons, from which they are transported into the stroma [7]. Further, these proteins are routed to the thylakoid or lumen by two main protein transport pathways in the thylakoid membrane. The secretory pathway (SEC) consists of the SecY/G channel and SecA protein, which utilize ATP hydrolysis and the proton motive force (PMF) to maintain proteins in an unfolded state and transport them into the thylakoid lumen [8,9,10,11]. A few of the proteins that are targeted via the Sec pathway are cytochrome, FTSH5, plastocyanin, and PSAF [12,13,14,15]. The other pathway, the Twin Arginine Transport pathway (TAT), can be found in plants, bacteria, and archaea. It only utilizes the PMF to translocate fully folded proteins across the thylakoid membrane [10,16,17,18]. The TAT system’s name comes from the conserved twin arginine motif (RR) found in the N-terminal signal peptide sequences of proteins that are specific substrates for TAT [10,19,20]. Some of the precursor proteins that are targeted to the TAT pathway are components of the oxygen-evolving complex PSII, PsbB and PsbQ, and subunit N of PS1 [21,22].

The cpTAT system mainly comprises three membrane-bound proteins: cpTatC, Tha4, and Hcf106. The bacterial homologs are TatC, TatA, and TatB, respectively [16]. The cpTatC protein is a 33 kDa protein with six linked transmembrane helices that span the thylakoid bilayer, resembling a glove-like structure [23,24]. The other two components, Hcf106 and Tha4, share a similar overall structure, consisting of an N-terminal transmembrane α-helix (TMH) linked to an amphipathic α-helix (APH) region via a hinge region, followed by an unstructured C-tail [16,25,26]. The functioning of the TAT system during active translocation is unclear. Mechanistically, the TAT system operates in a cyclic manner. In the resting state, cpTatC-Hcf106 and Tha4 form the heterotrimeric precursor receptor complex [27,28,29]. When a precursor protein with the twin arginine motif in the signal peptide binds to the receptor, Tha4 from the thylakoid membrane assembles into oligomers and attaches to the complex [30,31]. Following the oligomerization and assembly, the substrate protein’s mature domain is translocated in the presence of a PMF. A signal processing peptidase cleaves the signal peptide, allowing the mature protein to diffuse from the receptor complex to the thylakoid lumen [32,33]. The Tha4 (TatA) oligomers then disassemble from the receptor complex, resetting the (cp)TAT system for further substrate binding and transport [30].

**Figure 1:**
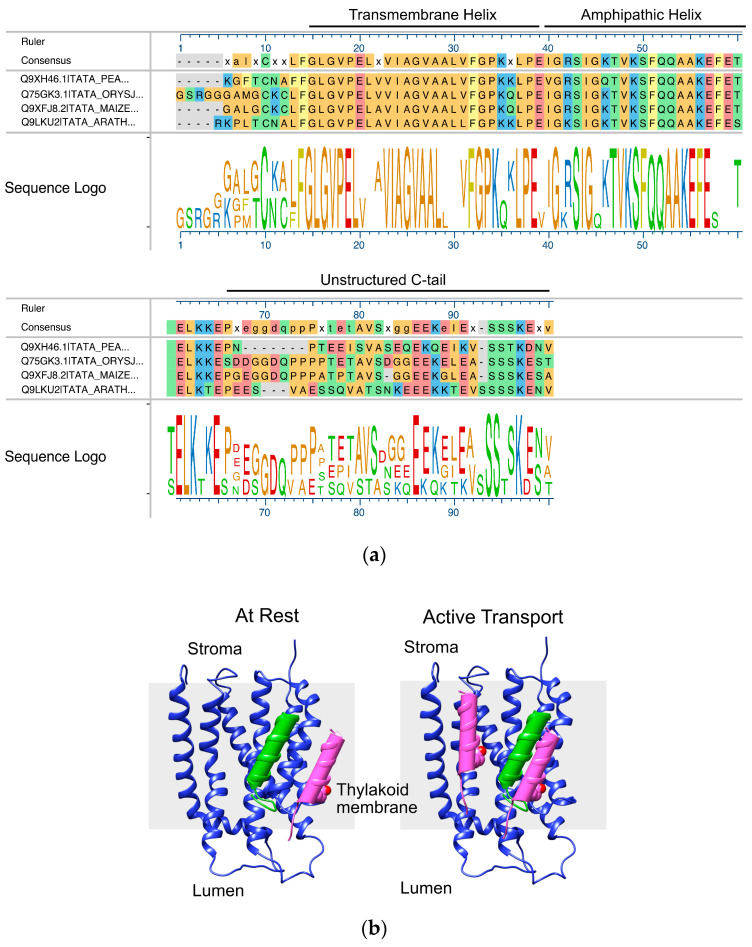
Tha4 has glutamate (E10) in the 10th position of the TMH. (**a**) Multiple sequence alignment and sequence logo plot of Tha4 from *Pisum sativum* (Uniprot: Q9XH46), *Arabidopsis thaliana* (Uniprot: Q9LKU2), *Zea maize* (Uniprot: Q9XFJ8), and *Oryza sativa* (Uniprot: Q75GK3), generated using MU SCLE alignment algorithm in MegAlign Pro™ (Version 15.3.0. DNASTAR, Madison, WI, USA). Amino acids are color-coded according to side chain chemistry (yellow = aromatic, red = acidic, blue = basic, orange = nonpolar, and green = polar) [16,37]. (**b**) cpTatC structural model by homology alignment between the final 230 residues from *P. sativum* (Uniprot: Q94G17) and the TatC crystal structure from *A. aeolicus* PDB: 4HTT [24]. Hcf106 TMH (green) was modeled on the TatB NMR structure from *E. coli* PDB: 2MI2 [40]. Alignment structures were created using Modeller v.10.7 [41] in UCSF ChimeraX 1.10.1 [42].

The glutamate at the 10th position in Tha4 is highly conserved among the plant Tha4 sequences (Figure 1a). Previous studies have found that glutamate at the 10th position of the Tha4 TMH is required for function in cpTAT-mediated precursor protein transport [34]. In this study, substituting glutamate with alanine (E10A) or glutamine (E10Q) failed to restore cpTAT transport, whereas an aspartate substitution supported transport, albeit to less than half the amount compared to the wild type, indicating the importance of a polar amino acid group at the TMH of Tha4 [34]. Substituting alanine for glutamate at position 10 also enhanced the resistance of Tha4 to alkaline extraction from the thylakoid membrane when treated with Na_2_CO_3_ and NaOH [34]. The oligomerization studies of Tha4 revealed that crosslinking between Tha4 monomers occurs with PMF and precursor during translocation. The study also demonstrated that Tha4 forms tetramers in unstimulated membranes and octamers in stimulated membranes by precursors and the proton gradient [30]. Likewise, the interactions between the amphipathic helix of Tha4 and the mature domain of the precursor lead to the stabilization of Tha4 oligomers [35]. These findings prompt further investigation into the formation of Tha4 oligomers and their role in active cpTAT translocation.

Currently, the exact role of transmembrane glutamate (E10) in Tha4 in the cpTAT-mediated translocation is unknown. Previous studies have shown that Tha4 undergoes a conformational change, causing the APH to tilt back toward the membrane, facilitating increased interaction with the APH [36]. In the same study, they found that the glutamate in the TMH is positioned in a more energetically favorable location, closer to the hydrophilic head groups. This positioning might allow for easier protonation by neutralizing the R carboxy group in the presence of a proton gradient during the Tha4 assembly with the receptor complex, enabling it to move into the hydrophobic membrane core to interact with the Transmembrane Helix (TM) 4 of the cpTatC (Figure 1b) [36,37]. In addition, the acidification of the environment surrounding the residue sensor during PMF formation could alter the ratio of protonated to deprotonated Tha4 glutamate residues. Such changes in this ratio may be associated with the Tha4 conformational shift observed experimentally during transport assays [35,36]. Consequently, voltage-gated ion channels exhibit analogous sensing functions, adjusting their conformations based on environmental changes PMF [38,39]. These findings led us to hypothesize that the Tha4 transmembrane glutamate may act as a sensor for promoting PMF in cpTAT-mediated transport. It is unclear that the glutamate at the 10th position requires a specific spatial or facial arrangement in the TMH of Tha4. This led to the question of whether moving this glutamate closer to the acidified lumen in alanine-substituted to Glutamate 10 in Tha4 variants could restore Tha4’s function.

In this study, we aimed to investigate how altering the hydrophobicity of the Tha4 TMH affects oligomer formation in the cpTAT translocon during active transport as opposed to unsimulated conditions, i.e., without the presence of PMF and functional precursor and as well as the position of a protonatable residue at the transmembrane helix related to the transport recovery. The in vitro translated Tha4 E10A, which had glutamate substitutions in the transmembrane region, could not complement cpTAT-mediated transport. However, aspartate mutations in the transmembrane region of Tha4 E10A provided weak complementing capacity for cpTAT function. Oligomer formation in the transmembrane helix depended on a functional precursor, while C-tail oligomerization responded mainly to the PMF. The amphipathic region showed no significant differences in oligomerization due to either precursor or PMF. Additionally, Tha4 oligomer formation increases with glutamate to alanine substitution but decreases with aspartate, altering interactions among Tha4 monomers. The data from the oligomerization profiles of each Tha4 region led us to model the Tha4 orientation in the membrane during the active cpTAT translocation.

## 2. Results

### 2.1. Tha4 Complementation Assays

#### 2.1.1. Sequential Glutamate Substitutions in the TMH of Tha4 E10A Failed to Complement the Loss of Function in Thylakoids Treated with αTha4 IgG

**Figure 2:**
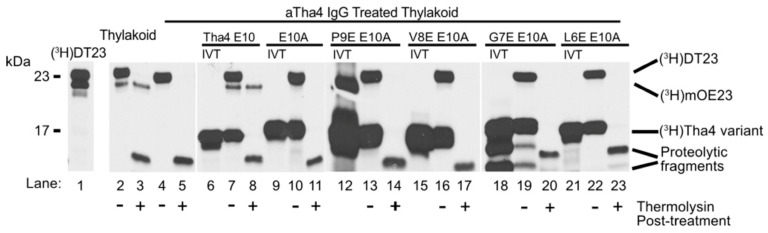
SDS-PAGE and gel fluorography results for glutamate-substituted Tha4 E10A variants. Radiolabeled DT23 (lane 1) was the precursor protein for the transport function assay; its maturation is shown by a size decrease. Mock thylakoid (lanes 2–3) served as a normal transport control, while other reactions used αTha4-blocked membranes (lanes 4–5, 7–8, 10–11, 13–14, 16–17, 19–20, 22–23). ‘IVT’ refers to in vitro translated, radiolabeled Tha4 single cysteine variants prior to thylakoid membrane integration. The + and − signs indicated whether the thermolysin treatment was applied or not.

To determine if the proximity of glutamate to the thylakoid lumen affects Tha4 function, we created several Tha4 variants with an alanine substitution for glutamate at the 10th position, along with additional glutamate or aspartate substitutions in the TM helix. Systematic mutations were introduced in the E10A background, substituting P9, V8, G7, or L6 with a glutamate residue (P9E, V8E, G7E, and L6E). These Tha4 variants were screened for their ability to restore transport of a modified precursor protein in the thylakoid membranes treated with αTha4 immunoglobulins (IgGs) against the endogenous Tha4. The precursor DT23 consists of a chimeric signal peptide linked to the 23 kDa mature domain of the oxygen-evolving complex of photosystem II protein (OE23, also known as PsbP), enhancing transport efficiency compared to wild-type preOE23 [43]. The functional transport of DT23 was confirmed by the appearance of a lower molecular weight band around 23 kDa in the lanes treated with thermolysin, a membrane-impermeable protease, in SDS PAGE and gel fluorography (Figure 2). Treatment of thylakoids with αTha4 IgGs inhibited transport (Figure 2 lanes 4–5), whereas endogenous cpTAT complexes in isolated thylakoid membranes facilitated the transport of DT23 (Figure 2, lanes 2–3). In the reaction samples where the in vitro translated Tha4 mutants were integrated into the intact thylakoid treated with αTha4 IgGs prior to transport, the cpTAT transport was restored (Figure 2 lanes 6–8). As expected, the Tha4 E10A variant was unable to restore transport (Figure 2 lanes 9–11) [34]. The substitution of glutamate in the transmembrane region of Tha4 E10A at the 9th through 6th positions (P9E, V8E, G7E, L6E) failed to restore DT23 transport, evidenced by the absence of a protease-protected mOE23 (Figure 2, lanes 12–23).

#### 2.1.2. Aspartate Substitutions in the TMH of the Tha4 E10A Variant Weakly Complement the Loss of Function in αTha4 IgG-Treated Thylakoids

**Figure 3:**
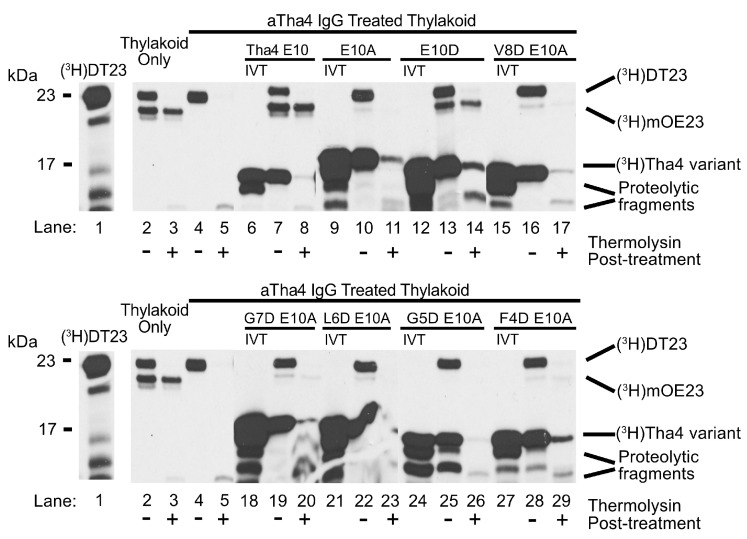
SDS-PAGE and gel fluorography results of glutamate-substituted Tha4 E10A variants. Radiolabeled DT23 (lane 1) served as the precursor protein used for the transport function assay; maturation of the precursor is indicated by a decrease in size. Mock thylakoid (lanes 2–3) acted as a transport control, while the remaining reactions were conducted with αTha4 blocked membranes (lanes 4–5, 7–8, 10–11, 13–14, 16–17, 19–20, 22–23, 25–26, 28–29). “IVT” corresponds to in vitro translated, radiolabeled Tha4 variants prior to their integration into the thylakoid membrane. Bands on the gel appearing below the size of recombinant Tha4 are likely proteolytic fragments following Thermolysin treatment.

Previous work has shown that substituting glutamate 10 with aspartate (E10D) weakly restores cpTAT transportation, whereas alanine substitution (E10A) fails to restore transportation [34]. We therefore questioned whether the acidic residue’s specific chemistry or its position in the TM influenced Tha4 transport functionality. To differentiate between these two factors, we conducted complementation assays using Tha4 E10A variants with aspartate substitutions sequentially from positions 8 to 4 along the TM. The transportation of the mature precursor DT23, is indicated by the lower band at approximately 23 kDa (Figure 3, lanes 2–3). Thylakoids treated with αTha4 Igs were unable to transport the precursor (Figure 3 lanes 4–5). The integration of wild-type Tha4 restored the transport of precursors, while Tha4 E10A could not complement cpTAT function. (Figure 3, lanes 6–11). As previously demonstrated, Tha4 E10D complements cpTAT transport of DT23 in αTha4-treated thylakoids, albeit with decreased efficiency compared to wild-type Tha4 integrated or isolated thylakoids (Figure 3, compare lanes 12–14 with 1–2 & 6–8). Interestingly, substituting aspartate in the 7th and 4th positions of the Tha4 E10A TM, transport was weakly restored (Figure 3, lanes 18–20 & 27–29). Transportation efficiencies for those variants were calculated using the method described in the Materials and Methods section, with efficiencies around 1% to 2%, while the E10D substitution showed about 10% (Figure S1). In contrast, aspartate substitutions at the 8th, 6th, and 5th positions did not restore the functional transport of the precursor (3, lanes 15–17 & 21–26).

### 2.2. Tha4 Oligomerization

**Figure 4:**
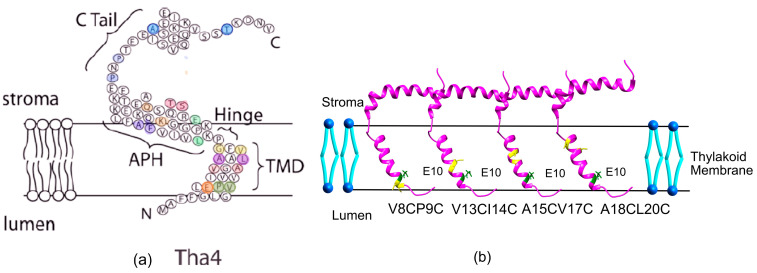
(**a**) The double cysteine variants selected for the crosslinking assays covering the TMH and hinge region are indicated by colored amino acid residues. (**b**) Double cysteine variants in the TMH utilized for crosslinking assays. The Protein Data Bank (PDB) structure of the *Arabidopsis* Tha4 (PDB ID 7B7O) was used to generate double cysteine mutants using UCSF ChimeraX 1.10.1 [42]. Green indicates Glutamate E10, while cysteine variants are highlighted in yellow.

After demonstrating that aspartate substitutions in the TM helix weakly restore transport, a question arose about whether these changes affected Tha4’s oligomerization during the active transport. To test the oligomerization of TMH, APH, and C-tail in Tha4 within the thylakoid membranes, we used a dual-cysteine crosslinking assay described previously [30]. We created a series of double cysteine mutants from the TMH, hinge, APH, and C-tail regions to use as crosslinkers in the crosslinking assays (Figure 4a). The functionality of the double cystine variants of Tha4 was investigated by analyzing their ability to integrate into the thylakoid membrane and their involvement in the TAT-mediated transport reaction. Complementation assays were utilized for testing the functionality of each variant according to the previously published protocol [34]. All the tested double cysteine variants were able to restore transport, and they were used in the following crosslinking assays (Figures S2–S6).

#### 2.2.1. Crosslinking Formation in the TMH Region Depends on the Presence of a Functional Precursor, Not on the PMF

For the investigation of TMH oligomerization, four sets of Cys-paired Tha4 variants were produced in the background of Tha4 E10, Tha4 E10A, and Tha4E10D, spanning the TMH of the Tha4 protein, including lumen-proximal, stromal-proximal, and membrane-buried residues (Figure 4b). The cross-linking experiment was carried out in the presence of a precursor and PMF [30]. For the crosslinking assays, the functional precursor (V-20F)tOE17 was utilized, which is the 17 kDa subunit of the oxygen-evolving complex. This precursor features a truncated transit peptide and includes a substitution of a phenylalanine at the -20 position (V-20F) in the cpTAT signal peptide region [44]. tOE17 has been used in many initial binding studies as a precursor, due to its superior binding and transport properties [45]. To dissipate the PMF, photophore/ionophore inhibitors such as Nigericin/valinomycin were added to the set of samples. Tha4-Tha4 interactions were mapped by incorporating radiolabeled Cys-substituted Tha4 into thylakoids, and catalyzing disulfide formation between nearby cysteine residues using a covalent, site-specific crosslinker for cysteine, Bismaleimidoethane (BMOE) [46]. To analyze the gel image obtained from the crosslinking assays, we employed ImageJ analysis to quantify the intensities of the gel bands (Figures S10–S13).

**Figure 5:**
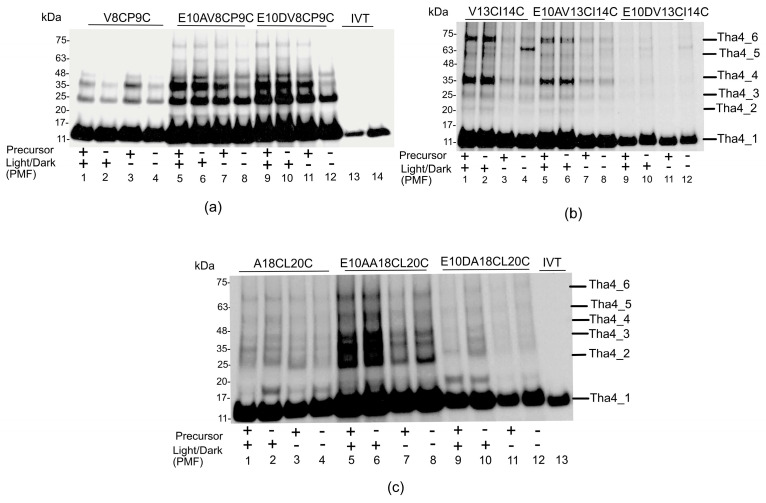
The formation of crosslinking in the TMH region relies on the existence of a precursor rather than on the proton motive force (PMF). (**a**,**b**) showed the V8CP9C and V13CI14C oligomer profiles, while the lower panel, (**c**) indicated A18CL20C. The crosslinking reactions were conducted with (+) and without (−) the functional precursor tOE17, which has a size of approximately 17 kDa. These experiments were performed under both light (+) and dark (−) conditions to investigate oligomer formation in relation to PMF availability. All the crosslinking reaction mixtures were normalized to equal chlorophyll concentrations mentioned in the Methods and Materials section.

The cysteines were positioned in the lumen proximal residue of the transmembrane domain, Tha4V8CP9C, a residue of key interest due to its close proximity to the transmembrane glutamate E10. Figure 5a illustrates that Tha4V8CP9C produced monomers with an average size of 13 kDa, which corresponds to the apparent molecular weight of fully reduced Tha4 containing double Cys residues in the TMH. This finding aligns with the previously reported crosslinking profile for V8CP9C [30]. The ladder’s step size is approximately 11 kDa, correlating with the 13 kDa Tha4 monomer, indicating that the ladder bands represent the Tha4 dimer, trimer, tetramer, pentamer, and hexamer, respectively (Figure 5). Tha4V8CP9C in the E10 background formed visible oligomers up to tetramers of ~50 kDa in size (Figure 5c lanes 1–4) under all the conditions. However, in the presence of a functional precursor, oligomers formed up to hexamers around ~75 kDa in size (Figure 5a lanes 1 and 3) and were intense than lanes 2 and 4, which do not contain a precursor (Figure S10a). The Tha4 double cys variants constructed under the aspartate and alanine background demonstrated oligomers up to hexamers with high-intensity bands regardless of the presence of a precursor or PMF (Figure 5a lanes 5–12). The oligomer profile of A15CV17C is similar to that of the V8CP9C variant, indicating that oligomer formation mainly responds to the presence of a functional precursor and PMF, but responds significantly in the presence of the precursor (Figures S7 and S10c).

For other residues (V13 and I14) buried within the thylakoid membrane, the formation of crosslinking on the Tha4 monomers resulted in higher oligomers of up to 6 monomers (Figure 5b). In the E10 double cys variants, more intense bands were observed in lanes 1 and 2 (Figure 5b and Figure S10b), where the crosslinking assays were performed in the presence of PMF. In the E10A variant, oligomer formation exhibited a similar pattern to the wild type, with a difference observed in lanes with PMF (Figure 5b lanes 5 and 6) compared to lanes without PMF (Figure 5b lanes 7 and 8). However, the aspartate-substituted double Cys Tha4 variants showed very few oligomers with V13CI14C.

Moving on to the stromal proximal residues in the TMH of Tha4 double cysteine variants, the crosslinking profiles for A18CL20C and E10DA18CL20C demonstrated a few crosslinks (Figure 5c). This observation agreed with the previously published data, where Tha4V17CL20C and L20CF22C failed to crosslink with each other [30]. Only the E10A A18CL20C variant showed oligomers up to tetramers in the presence of precursor and PMF, suggesting that the presence of the alanine (E10A) promotes oligomerization in a region where it does not occur natively (Figure 5c and Figure S10d). Even though A18 and L20 engaged in forming clusters, these residues are predicted to be oriented around one loop of the TM helix, not around a specific face of the helix [31,47]. Furthermore, this area is located close to where the protein exits the membrane, which may make it more flexible and capable of twisting compared to other sections of the protein, similar to observations made by scientists in other membrane proteins [48]. These factors may explain our observations, as other Tha4 variants in the TMH formed higher-order oligomer associations with neighboring monomers compared to Tha4 A18C L20C.

#### 2.2.2. In the Hinge Region, Oligomer Formation Does Not Affect the Presence of Precursor or PMF

**Figure 6:**
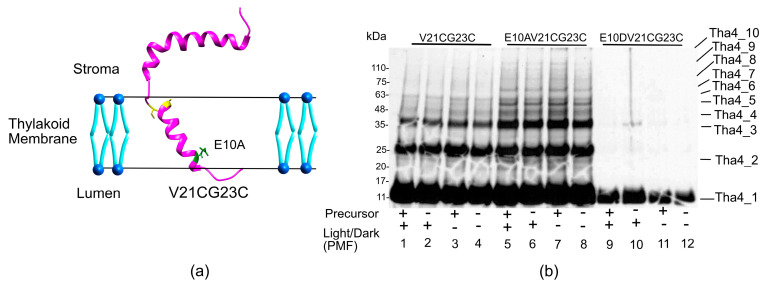
Oligomer profiles of the hinge region in the Tha4 protein. (**a**) Tha4 double cysteine residues selected for the crosslinking assay are modeled by the *Arabidopsis* Tha4 (PDB id: 7B7O) structure using UCSF ChimeraX 1.10.1 [42]. Green indicates Glutamate E10, while cysteine variants are highlighted in yellow. (**b**) The crosslinking reaction was performed on the double Cys Tha4 variant V21CG23C in the background of E10, E10A, and E10D, both in the presence (+) and absence (−) of a functional precursor and PMF. All the crosslinking reaction mixtures were normalized to equal chlorophyll concentrations before running on the 3–15% SDS gel as described in the Methods and Materials section.

Recent structural data on *Arabidopsis* Tha4 indicate that the TMH extends from amino acid residues V6 to L19 and the APH stretches from E27 to S48, together forming an L shape within the thylakoid membrane. The connector between the TMH and APH, referred to as the hinge region, spans amino acids F20 to P26 and holds lesser rigidity [47,49]. Given that the hinge region is short, its dynamics may be influenced by the two larger domains it connects. However, the relatively rigid dynamic behavior might also suggest a well-defined conformation [47,50]. The hinge region exhibited higher-order oligomers (Figure 6b). Under each reaction condition, E10A background, V21CG23C formed a ladder of oligomers up to a decamer (Figure 6b lanes 5–8), while V21CG23C showed up to 6 bands in the oligomeric analysis ladder (Figure 6b, lanes 5–8). The E10D variant followed a similar trend as the transmembrane cysteine variants, which were unable to produce many oligomers (Figure 6b lanes 9–12). The enhanced crosslinking formation between monomers in the hinge region might be due to the orientation of the hinge region, which is predicted to be outside the thylakoid membrane and facing toward the lumen, thereby allowing access to the other Tha4 monomers in the bilayer (Figure 6b and Figure S11) [47,49].

#### 2.2.3. The Amphipathic Helix Region, the Residues in the Stroma, Exhibited Greater Oligomer Formation in Both Wild-Type and Aspartate-Substituted Cysteine Mutants

**Figure 7:**
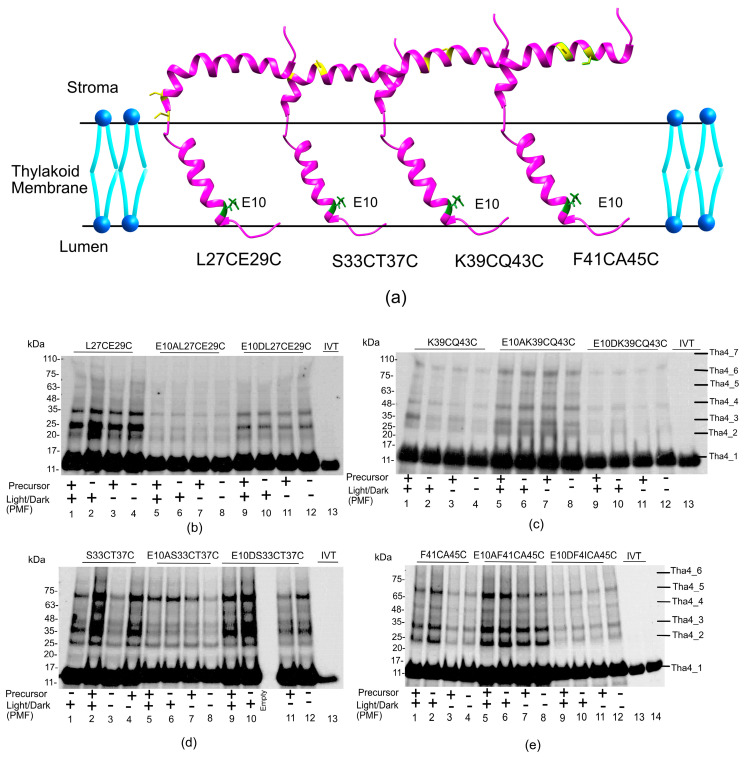
The amphipathic helix region, along with the stroma residues, showed increased oligomer formation in both wild-type and aspartate-substituted cysteine mutants. (**a**) Tha4 double cystine mutants were chosen from the APH region generated in *Arabidopsis* Tha4 (PDB id: 7B7O) using UCSF ChimeraX 1.10.1 [42]. (**b**,**c**) showed oligomer profiles of the L27CE29C and K39CQ43C, while (**d**,**e**) indicated the S33CT37C and F41CA45C. The crosslinking reactions were conducted as described in the Material and Method section. These experiments were conducted under both light (+) and dark (−) conditions and in the presence (+) and absence (−) of the precursor to investigate oligomer formation. All the crosslinking reaction mixtures were normalized to equal chlorophyll concentrations before running on the 3–15% Gradient SDS gel as described in the Methods and Materials section.

The APH region is either located on the thylakoid membrane or in the stroma of the chloroplast. Therefore, most amino acids in the APH region are highly accessible, allowing for crosslinking with other Tha4 monomers (Figure 7a). Tha4 double cysteine variants were selected spanning the APH region to investigate the crosslinking with each other. Overall, the oligomer formation in the APH region exhibited up to hexamers or heptamers in all the given conditions. The double cys variant E10 L27CE29C exhibited a more intense band compared to the E10A L27CE29C (Figure 7b lanes 1–4 vs. lanes 5–8 and Figure S12a). In the S33CT37C double cysteine variant, oligomer bands appeared more intense in lanes with a functional precursor and ΔpH (Figure 7d lanes 2 and 4) compared to lanes without the precursor and ΔpH (Figure 7d lanes 1 and 3 and Figure S12c). The E10A and E10D variants demonstrated oligomerization under all tested conditions without exhibiting a difference in the PMF or a functional precursor; however, enhanced intense bands were observed for lanes 9 and 10, which were supplied with PMF, compared to lanes 11 and 12 without PMF in Figure 7d in the E10D S33CT37C variant.

Double cysteine substitutions at the K39 and Q43 showed no apparent difference in oligomerization pattern in the presence of PMF and light (Figure 7c lanes 1–4). E10AK39CQ43C variant demonstrated enhanced crosslinking compared to the aspartate and wild-type variants, suggesting that a greater number of Tha4 oligomers is formed in the alanine substitution variants (Figure 7c lanes 5–8 and Figure S12b). In the E10DQ36CK39C variants, oligomers were less likely to form than that of the wild type and the alanine-substituted double-cys variants and only exhibited oligomers up to tetramers (Figure 7c lanes 9–12).

In the F41CA45C variant, oligomer formation was enhanced in the presence of a PMF (Figure 7e lanes 1 and 2 and Figure S12d) compared to the reactions where the PMF was dissipated (Figure 7e lanes 2 and 4). Bands in the E10A F41CA45C exhibited Tha4 oligomer formation up to hexamers, similar to the wild type. However, crosslinking was enhanced in all conditions, indicating a denser oligomer formation. In the aspartate substitutions, crosslinking was similar to the wild-type E10, although no response to the presence of PMF was observed.

According to the NMR data, the N-terminal residues of the APH are deeply buried in the micelles, this suggests more closely packed Tha4 monomers, shielding them from the solvent, which leads to tight packing and ultimately results in enhanced crosslinking between neighboring cysteine residues, as seen in the L27CE29C and S33CT37C (Figure 7b,d). Since the C terminus of the APH is predicted to be more solvent-exposed, the orientation of the cysteine-substituted amino acids might be further apart, resulting in fewer crosslinks and making oligomers as observed in K39CQ43C and F41CA45C (Figure 7c,e) [47].

#### 2.2.4. Oligomer Formation in the C Tail Mainly Responds to the Presence of a PMF, Regardless of the Presence of the Precursor

The C-tail of Tha4 is thought to be unstructured and resides in the stroma of the chloroplasts [25,47]. Therefore, the crosslinking formation in the C-tail was observed to be undefined due to its highly dynamic nature. The P56 and P59 double cys variant showed oligomers up to tetramers, and oligomer formation was not affected by the presence of a functional precursor or a PMF in all wild-type, alanine, and aspartate substitutions (Figure S8).

**Figure 8:**
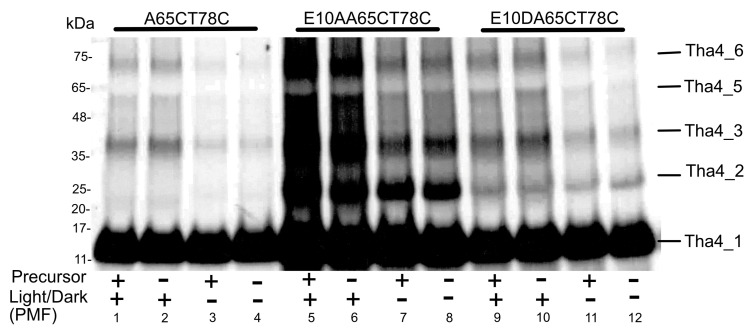
The oligomer profiles of the Tha4 double cys variants in the C-tail. Crosslinking reactions for A65CT78C was conducted as described in Material and Methods. These experiments were performed under both light and dark conditions, in the presence (+) and absence (-) of the precursor, to investigate oligomer formation. All the crosslinking reaction mixtures were normalized to equal chlorophyll concentrations before running on the 3–15% Gradient SDS gel as described in the Methods and Materials section.

The A65CT78C variant, located near the end of the C-tail, exhibited oligomers up to pentamers and a distinct response to the PMF, regardless of the presence of the precursor. Lanes 1 and 2 with the PMF displayed enhance crosslinking compared to lanes 3 and 4, where the PMF is dissipated (Figure 8 and Figure S13b). The same trend was noted in the E10A variants (Figure 8, lanes 5 and 8). The aspartic acid substitution in the A65CT78C also demonstrated a difference in the crosslinking formation due to the PMF (Figure 8, lanes 9 and 10).

## 3. Discussion

Plant Tha4 protein is crucial for cpTAT-mediated translocation, thought it serves primarily as the point of passage for the precursor. Prior research has shown that Tha4 is present in a molar excess compared to cpTatC and Hcf106, and it is engaged in the resting and translocation stages [16,29,44]. Furthermore, it has been found that, upon stimulation by the presence of the precursor and the PMF, additional Tha4 assembles into the receptor complex by forming higher-order oligomers [31] and during translocation, Tha4 directly interacts with the mature domain of cpTAT precursors [35]. Most importantly, Tha4 undergoes a conformational change in response to the precursor, exposing more of the amphipathic helix to the stroma [36]. However, it was unclear from these prior studies how Tha4 could sense the presence of the PMF to undergo the changes reported. In this study, we aimed to investigate the possibility of glutamate acting as a sensor for the PMF, thereby initiating the conformational change in the Tha4 protein by analyzing the oligomer formation in various regions of Tha4 in the presence of a functional precursor and PMF.

The side chain of glutamate features a terminal carboxylic acid group (–COOH) that typically has a pKa of approximately 4.0–4.5 in an aqueous solution giving it a negative charge at physiological pH (~pH 7.4) [51,52,53,54]. The protonation state of its γ-carboxyl side chain depends on the local pH. Since E10 is in close proximity to the acidified lumen (approximately pH 5.8–7) during transport, it is likely that this residue exists in a protonated form [55,56]. In synthetic peptides, model bilayers, or when it is buried within the hydrophobic core of a protein, experimental pKa values range from about 6 to 9 [52,57]. This environmental increase in the pKa of the γ-carboxyl side chain may promote the protonation of Glu10, altering its charge state.

The presence of PMF is crucial for recruiting Tha4 to the cpTAT complex, potentially altering the ratio of deprotonated to protonated Tha4. This alteration might lead to a subpopulation that undergoes the noted topological shift and organizes at the receptor complex with the precursor bound [36,37,58]. Previously, molecular modeling also demonstrated that the oligomeric structures of TatA in *E. coli*, with Gln8 oriented inward towards the TatA pore, facilitate engagement with water. Then, this pore distorts the lipid bilayer, creating a pathway for protein translocation while minimizing ion leakage [58]. Additionally, the same study found that TatA monomers adopt a tilted position in the membrane to accommodate their short transmembrane helix (TMH) [58]. Similar to the TatA model, a topological shift in Tha4 was observed during transport, indicating that E10 is exposed in the lumen. This could enable protonation of the TMH glutamate γ-carboxyl side chain when the membrane is energized by a proton gradient, which is essential for Tha4 to assemble with the precursor-bound receptor complex [36]. Similar sensing capabilities have been demonstrated for voltage-gated ion channels, which change their conformations in reaction to alterations in the PMF [38,59,60,61,62].

### 3.1. Restoration of cpTAT Transport Defect by TMH Charge Variants of Tha4 E10 in IgG-Treated Thylakoids

Based on the complementation assay data, we conclude that the proper positioning of glutamate 10 in the TMH of Tha4 is required for functional transport. (Figure 2 and Figure 3). The results from the complementation assays showed that the substitution of an aspartate at the 4th or 7th residues weakly restores Tha4 E10A function (Figure 3, lanes 18–20 & 27–29). Aspartate substitutions in the 5th, 6th, and 8th positions of Tha4 E10A were unable to restore function and transport (Figure 3, lanes 15–17 & 21–26). One possible reason for these findings is that Tha4’s protonation state affects its stability in the membrane. Tha4 E10A has increased stability in the thylakoid membrane, as it has been shown to be significantly less susceptible to alkaline extraction compared to E10 and E10D variants [34]. Aspartate substitutions in Tha4 E10A would subsequently result in reduced inherent stability in the thylakoid membrane by reintroducing a polar residue into the hydrophobic transmembrane domain (Figure 3). The stability of the TM helix in the thylakoid membrane could be altered in response to protonation and deprotonation events that arise from changes in the local pH during the generation of the PMF. (Figure 1b).

### 3.2. Crosslinking Formation of Tha4

Based on the disulfide crosslinking observed between Tha4 monomers in this study, which can be categorized into four main observations: oligomer formation in response to the functional precursor, oligomer formation in response to PMF, oligomer formation that occurs regardless of PMF or precursor, and oligomer formation dependent upon both PMF and precursor. Tha4 oligomer formation is found to be in response to both the PMF and a functional precursor [30,31]. However, oligomer formation in the TMH region occurs in response to the presence of precursor rather than the PMF (Figure 5a). These data further suggest that the TMH with native glutamate plays an essential role in protomer interaction during the active TAT translocation, which may be by interacting with the mature domain of the precursor [34,35]. The V8CP9C variant was previously assessed for oligomerization due to its proximity to glutamate E10 in the TMH [30]. In that investigation, Tha4V8CP9C formed tetramers in unstimulated membranes, and with the addition of a PMF and precursor, oligomers reached sizes up to octamers [30]. However, in this study, the V8CP9C variant exhibited oligomers up to hexamers in similar conditions (Figure 5a). The primary reason for the difference in the number of oligomers formed can be attributed to the use of a crosslinker. In a previous study [30], they employed Cu2+-phenanthroline, an oxidative zero-length cross-linker, that promotes reversible disulfide bond formation [63]. In this study, we used a homobifunctional thiol crosslinker, BMOE, with a spacer arm of 8 Å in length, which tends to produce fewer and more specific crosslinks compared to Cu^2+^-phenanthroline [64]. Evidence shows BMOE is a selective crosslinker, promoting specific dimers and reducing higher-order oligomerization. This makes it beneficial for studies needing controlled crosslinking [64,65,66].

By substituting the TMH glutamate residue with alanine, a small, nonpolar amino acid without an ionizable side chain, we can effectively remove both the charge and the ability for pH-dependent protonation or deprotonation at that site. This strategy enables us to differentiate between the effects of the residue’s side chain chemistry and its protonation state on driving or stabilizing oligomer formation. In our crosslinking studies, oligomer formation can be observed in the E10A-substituted double-cys variants throughout the Tha4 protein. However, no significant difference in the crosslink formation can be seen in response to either PMF or precursor, except for two variants: V13CI14C and A65CT78C in the TMH region and the C-tail, respectively. If oligomerization still occurs despite the alanine substitution, it suggests that the specific side chain (and its dynamic charge state) is not strictly required for subunit assembly. However, we cannot completely ignore the fact that in complementation studies, the E10A variants failed to restore the cpTAT-mediated transportation [34]. We can assume that there are two pools of Tha4 within the membrane: one bound to the receptor complex and another existing in a separate pool. In order to elucidate this scenario further, one may assume that, despite the formation of oligomers in the E10A variants in the free Tha4 pool, these E10A variants would be incapable of undergoing the conformational shift, thereby not engaging in TAT transport. This is likely due to several factors. Substituting a hydrophobic, nonpolar residue stabilizes the TMH of Tha4 within the membrane, as evidenced by the enhanced insensitivity of Tha4 E10A during an extraction [34]. Furthermore, the Eisenberg hydrophobicity values for the E10A double cysteine variants are higher than those of the wild type and E10D variants (Figure S9) confirming the greater stability of the E10A variant in the membrane. Therefore, E10A variants might stabilize the TMH and arrange themselves in the membrane to allow for tighter packing among the neighboring Tha4 monomers, leading to tighter helix-helix interactions. This configuration of E10A could inhibit the dissociation of Tha4 monomers from the oligomers in the thylakoidal separate pool, ultimately blocking their association with the precursor-bound receptor complexes [27,28,37,67].

### 3.3. Hydrophobic Mismatch and Tha4 TMH Hydrophobicity

**Figure 9:**
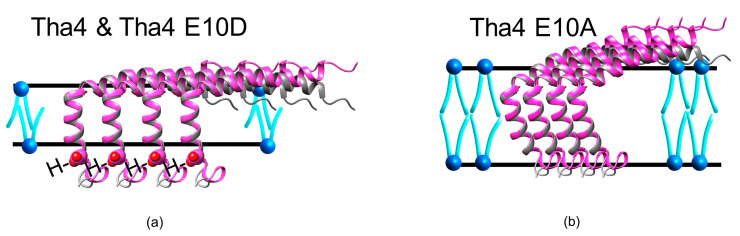
(**a**) Model of Tha4 E10/D oligomerization during precursor transport. (**b**) Model of Tha4 E10A oligomerization in the thylakoid membrane. Tha4 (pink) primary sequence residues 55–137 from *P. sativum* (Uniprot: Q9XH46) were modeled onto the solution NMR structure of TatA d from *B. subtilis* (PBD: 2L16) [51] The Tha4 conformational change and the proposed compression of the thylakoid membrane due to hydrophobic mismatch during PMF generation are illustrated with cartoon lipids. Alignment structures were generated using Modeller v.10.7 [41] in UCSF ChimeraX 1.10.1 [42].

Another aspect that needs to be considered regarding the Tha4 protein is the hydrophobic mismatch and the TMH hydrophobicity. In bacteria, primarily, the transverse hydrophobic environment in the cytoplasmic membrane is about 3 nm [68]. In contrast, the bacterial TatA TMH region is predicted to contain only 15 residues, approximately 1.8 nm in length [58]. In plant thylakoids, the thylakoid membrane measures about 3.2 nm to 4.3 nm under light and dark conditions, respectively [69], and the Tha4 TMH is composed of ~12 amino acids, about 1.4 nm, which does not have enough length to span the membrane with only the transmembrane helix. This discrepancy results in a hydrophobic mismatch and destabilization of the membrane, and potential destabilization of the membrane, which may promote thinning and facilitate the translocation of precursor proteins through the thylakoid membrane [70,71,72,73]. Studies have shown that the hydrophobic length of the TMH influences its orientation in TMH in bacterial TatA, where the TMH adopts a higher tilt angle. Positive hydrophobic mismatch, for example, causes a transmembrane helix to tilt more, while a negative hydrophobic mismatch results in aggregations [50]. Therefore, in E10A substitutions, the orientation of Tha4 monomers might adopt different angles due to the proposed tighter packing. This could hinder substrate translocation by disrupting proper Tha4 oligomer formation in the existing molecular models of cpTAT function (Figure 9b) [16].

According to recent studies on the structures of Tha4 oligomers in solution and in the membrane systems, it was found that the hydrophobic helices embed in the membrane core, while the residues from amphiphilic helices align at the membrane surface facing the center of the oligomers [49]. Our crosslinking data obtained in the presence of precursor and PMF during the active transport support the predicted orientation of the APH, where the residues closer to the N terminal part of the APH showed enhanced crosslinking compared to the C terminal region, indicating the closer orientation in the Tha4 monomers through the beginning of the N terminus of APH. These data agree with the previous results, which revealed that in the transport state, the APH is thought to partition more uniformly and deeply into the membrane via the N-terminal region, compared to a less membrane-active state under resting conditions. This conformational shift arises from the deeper insertion, likely contributing to the membrane destabilization needed for the translocation of folded proteins [36]. Direct evidence of PMF-driven membrane thinning in the TAT system is limited. However, thylakoids demonstrate clear PMF-dependent thinning, likely driven by the protonation of membrane components. These findings support the notion that the Tat system may utilize PMF-driven local membrane thinning to facilitate protein transport [74,75,76].

However, it is interesting to see that, in the E10A background, double cysteine variants closer to the C terminus of the APH show enhanced crosslinking compared to the N-terminal residues of the APH. The absence of Glu10 may hinder PMF sensing and proper orientation in the translocon. Additionally, tightly packed N-terminal residues could restrict BMOE access, reducing crosslinking efficiency compared to the more accessible C-terminal region.

The C-tail region of the Tha4 and TatA is predicted to be unstructured and highly dynamic in nature [25,77]. Previous crosslinking studies conducted on the A65CT78C double cysteine variant, in the presence of the precursor and PMF, demonstrated that the depletion of PMF in the presence of twin arginine containing signal peptide with tpOE17 resulted in fewer crosslinking interactions between adjacent Tha4 C-tails [30,31]. Our crosslinking data also aligns with the previous results; however, our data exhibit a significant difference in oligomer formation in response to the PMF rather than to the precursor (Figure 8). The same trend was observed in the E10A and E10D A65CT78C variants. The direct relationship between the C-tail of Tha4 or TatA with TAT translocation has not yet been discovered. However, the conformational changes occurring in the TMH and APH in Tha4 could bring C-tails into close, tight contact to form oligomers, creating a dense mesh surrounding the substrate protein [30].

The PMF is found to be crucial for the assembly of TatA and for the translocation of precursors through the TAT system through mechanisms that involve protonation-induced conformational changes and the energetics of membrane potential-dependent assembly [33,36,58]. Residues in the C terminus of the APH and C tail (Figure 7d and Figure 8) exhibited enhanced oligomer formation in response to the PMF than the precursor. The exact mechanism by which PMF drives oligomerization in the TAT translocon remains unclear; however, studies indicate that protonatable residues, such as histidine in the TMH, can act as pH sensors and induce conformational changes in membrane proteins [78]. Similar to such systems, glutamate at the TMH may function as a pH sensor, as observed in the oligomerization response to PMF in the C tail and some residues in APH.

### 3.4. Proposed Model for Tha4 Oligomer Assembly

Oligomer formation in the E10D variant of Tha4 did not yield notable crosslinks; however, complementation studies revealed that E10D partially restored transport of the DT23 precursor [34]. The differences in the side chains of aspartate and glutamate may affect Tha4 monomer assembly at the TAT translocon and consequently transport efficiency. Nevertheless, the partial recovery observed suggests that some E10D monomers can still assemble at the translocon. The pKₐ of the β-carboxyl side chain of aspartate is approximately 3.4 in aqueous solution; therefore, at physiological pH (~7.4), the aspartate side chain is negatively charged, similar to glutamate [79]. During transport, where the lumen pH ranges from 5.8 to 7, aspartate is likely to be protonated [55,56]. Both aspartate and glutamate carboxyl chains share similar characteristics, and the oligomer formation in the APH region displayed comparable patterns for both E10D and native E10, suggesting that some of the E10D Tha4 variants may still engage in oligomerization, likely due to the protonation capacity of aspartate. Conversely, most of the E10D variants would remain in the separate free pool Tha4, having tight interactions with monomers, and would be unable to assemble in the receptor complex (Figure 9a).

**Figure 10:**
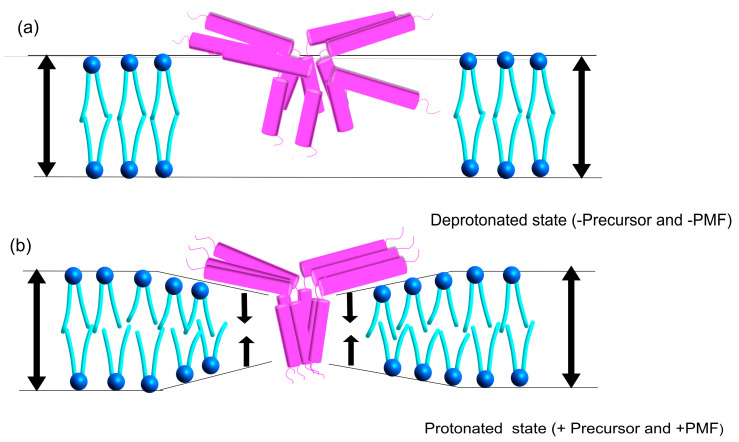
Proposed model for Tha4 assembly in the protonated state and the deprotonated state in the thylakoid membrane. In its deprotonated state, we can expect Tha4 to assemble in the membrane in a disorganized way and interact with the helix-helix interface of the adjacent Tha4 TMH. APH of the Tha4 would lie on the membrane as seen in the previous studies [77]. When a precursor and PMF are present, Tha4 organizes more systematically, as evidenced by the crosslinking data. TMH tends to form parallel bundle-like interactions. Meanwhile, APH positions themselves nearer to each other, with their N-terminal regions facing one another. This conformational shift would lead to the membrane thinning and translocation of the precursor through the cpTAT pathway. Arrows depict the thylakoid membrane thickness in the protonated and deprotonated state.

The organization of Tha4 oligomers in the receptor complex and the proposed conformational shift that arranges Tha4 oligomers during cpTAT translocation have yet to be defined. Our results demonstrated that Tha4 V8C P9C formed higher-order oligomers than Tha4 A18C L20C in the presence of precursor and PMF (Figure 5a,c). This observation can be interpreted as the interactions between neighboring Tha4 E10 helices transitioning from a “crossed point” configuration to a parallel, side-by-side arrangement as Tha4 bundles gather at receptor complexes bound to precursors. Our crosslinking data support the observed shift in the TMH, as other residues tested in this region also exhibited oligomer formation, primarily in response to the precursor, in the presence of PMF. We further propose that the APH region undergoes a conformational change, likely in response to the protonation change in the glutamate and the conformational change observed in the TMH. Notably, the N-terminal amino acid residues in the APH showed greater crosslinking than the C-terminal region, suggesting a shift that pulls Tha4 closer into the membrane during translocation, contributing to membrane thinning (Figure 10) [16,36,49,58]. This study further highlights the importance of glutamate E10 in the TMH for cpTAT translocation. To further investigate whether transmembrane glutamate (E10) could act as a proton or voltage-sensitive residue initiating conformational changes during cpTAT activation, future studies combining biochemical and electrophysiological techniques, such as reconstituting Tha4 into artificial lipid bilayers or proteoliposomes for voltage-clamp analysis, may allow for a quantitative assessment of its gating behavior and response to the proton motive force [80,81]. Furthermore, to understand the connection between pH, cpTAT transport, and Tha4 oligomers, it is essential to examine how Tha4 oligomers respond to the PMF and their interactions with other TAT components in future studies.

## 4. Materials and Methods

### 4.1. Chloroplast and Thylakoid Isolation

According to the published protocol, chloroplasts were isolated from 10- to 12-day-old *Pisum sativum* (pea) plants [30,82]. In brief, dwarf peas were grown in coarse-grade vermiculite for 12–14 days at 20 °C, under 150 μE of fluorescent light, 16 h day/8 h night in a growth chamber. Leaves from the young pea plants were cut and homogenized in 250 mL of buffer containing 50 mM HEPES/KOH, pH 7.5, 0.33 M sorbitol, 1 mM MgCl_2_, 1 mM MnCl_2_, 2 mM EDTA, 5 mM Na-ascorbate, and 1% BSA. Intact chloroplasts were isolated via a Percoll density gradient (lower band). Intact chloroplasts were suspended in an import buffer (IB) containing 50 mM HEPES-KOH (pH 8.0) and 300 mM sorbitol, which helps create an osmotic environment and maintain the integrity of the thylakoid membranes. The concentration of chlorophyll was then determined to be 1 mg/mL by measuring the UV-Vis absorbance at 663 nm and 645 nm [83]. Thylakoids were isolated from intact chloroplasts according to the previously described procedure [80]. In short, isolated intact chloroplasts were suspended in hypotonic lysis buffer containing 20 mM HEPES-KOH, pH 8, and 10 mM MgCl_2_ on ice for 10 min, then centrifuged at 3000× *g* for 8 min. The supernatant was further centrifuged at 13,000× *g* for 60 min to obtain the stromal extract, which was used to dilute the precursor protein for in vitro transport assays. Pelleted thylakoids were resuspended to 1mg/mL chlorophyll in IB containing 10 mM MgCl_2_ and stored on ice for downstream applications and assays.

### 4.2. Generation and In Vitro Translation of Tha4 Double Cys Variants and Precursor DT23, (V-20F)tOE17

Single and double cystine substitutions in Tha4 E10/A/D were generated using primer-based site-directed mutagenesis polymerase chain reaction (PCR) with Phusion polymerase, following the manufacturer’s instructions. (New England Biolabs, Ipswich, MA, USA). Tha4 single and double Cys variants were radio labeled by the [^3^H] Leucine (Leu), then the in vitro translation was carried out by the Promega Wheat Germ Extract translation kit (Madison, WI, USA) with capped mRNA [3]. Translation reaction mixtures were mixed with an equal volume of 60 mM leucine in 2x IB and kept on ice until needed. The precursor proteins DT23, which is an oxygen evolution protein 23 kDa with a modified signal peptide and (V-20F)tOE17, which is a modified variant of the 17 kDa oxygen-evolving complex protein with phenylalanine replacing the valine at the 20th residue before the mature OE17’s first amino acid, were also translated in vitro using the Promega wheat germ kit. They were then diluted with an equal amount of extracted stromal extract to achieve a final dilution of 1:4.

### 4.3. Substituting Endogenous Tha4 and Complementing cpTAT

The functionality of the Tha4 E10A glutamate and aspartate TMH single cysteine and double cysteine variants was assessed using an in vitro complementation assay published previously [34]. To summarize, endogenous Tha4 was inhibited in isolated thylakoids (1 mg/mL) through treatment with purified anti-Tha4 immunoglobulin G (IgG), followed by treatment with Staphylococcus aureus protein A (Sigma-Aldrich, St. Louis, MO, USA). The in vitro-translated Tha4 variants were incorporated into αTha4-treated thylakoids by incubating them for 20 min at 15 °C. Following this, thylakoids were washed and recovered for subsequent testing of transport efficiency. The in vitro-translated DT23 precursor protein is transported via the cpTat system under illumination conditions (approximately 100 μmol/m^2^/s) at 15 °C for 15 min. Transport reactions were stopped by placing samples on ice. The thylakoids were then retrieved and treated with thermolysin to degrade any non-transported DT23. The resulting protein samples were analyzed using SDS-PAGE and gel fluorography, following the methods described below.

### 4.4. Disulfide Crosslinking Assay

Disulfide crosslinking assays were conducted according to the previously published protocol [3]. Isolated thylakoids were suspended in 1x IB with 0.5 mM DTT and incubated with freshly prepared 2.5 mM ethylmaleimide (NEM) in 95% EtOH to block the endogenous cystine residues. NEM treatment was terminated using three times the volume of 10 mM DTT in 1x IB. Thylakoids were collected through centrifugation and then resuspended to their original volume with IBM. Radiolabeled in vitro translated double-cys Tha4 variants were integrated into the NEM treated thylakoids by incubating for 20 min at 15 °C. Double cys-substituted Tha4 integrated thylakoids were recovered by centrifugation and washed with excess 1x IBM. Individual crosslinking reactions included 50 μM ATP, 0.1 mM DTT, and 3.3 mM MgCl_2_. Reactions that dissipated the PMF also included 0.5 μM nigericin and 1 μM valinomycin. The transport reaction was initiated by adding either 30 μL of in vitro translated (V-20F)tOE17 at a final concentration of 1.5 μM, or 1x IB as a control. The light-induced transport reaction mixtures were then incubated at 15 °C for 5 min in a circulating water bath under approximately 100 μmol/m^2^/s of white light illumination from a halogen lamp, while the dark-induced transport reaction mixtures were incubated on ice, subjected to dark conditions. The oxidative crosslinking reaction was initiated by adding 0.25 mM Bismaleimidoethane (BMOE) and allowing it to proceed for 10 min in the water bath. The crosslinking reaction was quenched by adding 5 times the excess of IB and 14 mM EDTA. Thylakoids were recovered by centrifugation and normalized to equal chlorophyll concentrations according to the previously published protocol by adding reducing and non-reducing buffers to achieve the same chlorophyll concentration in all reaction samples [81]. Each disulfide crosslinking assay was performed 3 times.

### 4.5. SDS-PAGE and Fluorography

Samples from the complementation assays and half of the samples from crosslinking assays were dissolved in a 2X non-reducing sample buffer containing 100 mM Tris-HCl (pH 6.8), 8 M urea, 5% SDS, 30% glycerol, and 0.1% bromophenol blue. The other half of the crosslinking assay samples were dissolved with a 2X reducing sample buffer containing beta-mercaptoethanol. The samples were analyzed using 5–13.5% gradient Tris-Tricine SDS-PAGE and gel fluorography. In summary, 12% gels or 5–13.5% gradient Tris-Tricine gels (for crosslinking assays) were prepared for fluorography by incubating in DMSO (ThermoFisher Inc., Waltham, MA, USA) to enable the internal deposition of 2,5-diphenyloxazole (ACROS). These were then dried on filter paper and exposed to X-ray film (Carestream Health Inc., Rochester, NY, USA). ImageJ v.154p software (developed by the National Institutes of Health (NIH)) was used to measure band intensities for analyzing the gel images.

## Data Availability

The raw data supporting the conclusions of this article will be made available by the authors on request.

## Supplementary Materials

Supporting information can be downloaded at: https://www.mdpi.com/article/10.3390/plants14213338/s1, Figure S1: Tha4 E10D variants differ in their ability to restore transport. Figure S2: The Tha4 double cysteine variants S33CT37C and A15CV17C restore cpTat transport. Figure S3: The Tha4 double cysteine variants V13CI14C and F41CA45C restore cpTat transport. Figure S4: Tha4 double cysteine variants V21CG23C and A18CL20C restore cpTat transport. Figure S5: The Tha4 double cysteine variants L27CE29C, V8CP9C and K39CQ43C restore cpTat transport. Figure S6: The Tha4 double cysteine variants A65CT78C, P56CP58C, E10D K39CQ43C and E10DL27CE29C restore cpTat transport. Figure S7: The formation of crosslinking in the A15CV17C relies on the existence of a precursor rather than on the proton motive force (PMF). Figure S8: The formation of crosslinking in the P56CP58C in the C-tail of the Tha4. Figure S9: Eisenberg hydrophobicity values of residues 5–30 Tha4 in cysteine-free and double-cysteine Tha4 E10/A/D variants. Figure S10: Crosslinking formation in the TMH region quantified using ImageJ. Figure S11: Crosslinking formation in the hinge region, V21CG23C quantified by using Image J. Figure S12: Crosslinking formation in the APH region quantified by using Image J. Figure S13: Crosslinking formation in the C tail region quantified by using Image J.

## Abbreviations

The following abbreviations are used in this manuscript:

cpTAT	Chloroplasts Twin Arginine Transport
PMF	Proton Motive Force
Cys	Cysteine
TMH	Transmembrane Helix
APH	Amphipathic helix
TM	Transmembrane
E10	Glutamate at the 10th position
E10A	Glutamate substituted with alanine
E10D	Glutamate substituted with aspartate
E10XC	Wild type with glutamate substituted with single cysteine variants
E10A XC	Glutamate substituted with alanine with single cysteine variants
E10D XC	Glutamate substituted with aspartate with single cysteine variants
E10XCYC	Wild type with glutamate substituted with double cysteine variants
E10A XCYC	Glutamate substituted with alanine with double cysteine variants
E10D XCYC	Glutamate substituted with aspartate with double cysteine variants
