# Supplementary material for: The Transmembrane Glutamate Serves as a pH Sensor for Tha4 Oligomerization During Twin Arginine Transport of Proteins
Source: Plants (Basel). 2025 Oct 31;14(21):3338. doi: 10.3390/plants14213338 (PMC12609122; doi:10.3390/plants14213338)
Supplement: Supplementary file 1 [file plants-14-03338-s001.zip › plants-3748481-supplementary.pdf]

plants

Article

The transmembrane glutamate serves as a pH sensor for Tha4 oligomerization during Twin Arginine Transport of proteins

Vidusha S. Weesinghe[1], Christopher Paul New[2] and Carole Dabney-Smith[1,*]

[1]Department of Chemistry and Biochemistry, Miami University, Oxford, Ohio 45056, The United States
[2]Cell Molecular Structural Biology Graduate Program, Miami University, Oxford, Ohio 45056, The United States
*Correspondence: smithac5@miamioh.edu; (+1)513-529-8091

Supplemental Figures

Tha4 complementation assays for investigating the functionality of cysteine mutants.

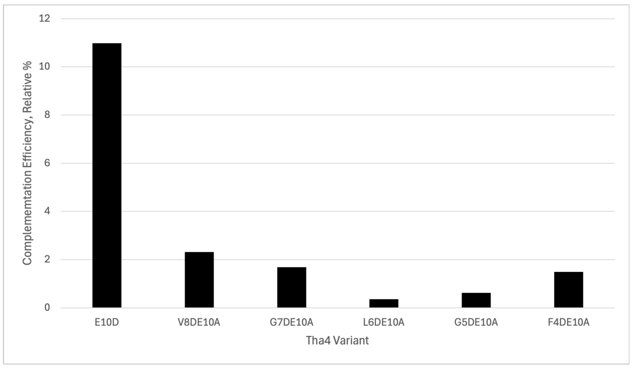

Figure S1. Tha4 E10D variants differ in their ability to restore transport. Complementation efficiency was calculated by normalizing the transported DT23 in anti-Tha4–treated thylakoids to the relative amount of recombinant Tha4 integrated into the thylakoids and then compared to transport of the precursor in the presence of wild-type Tha4. Transport of precursor in the presence of antibody-treated thylakoid was 0%. See Materials and Methods for additional information.

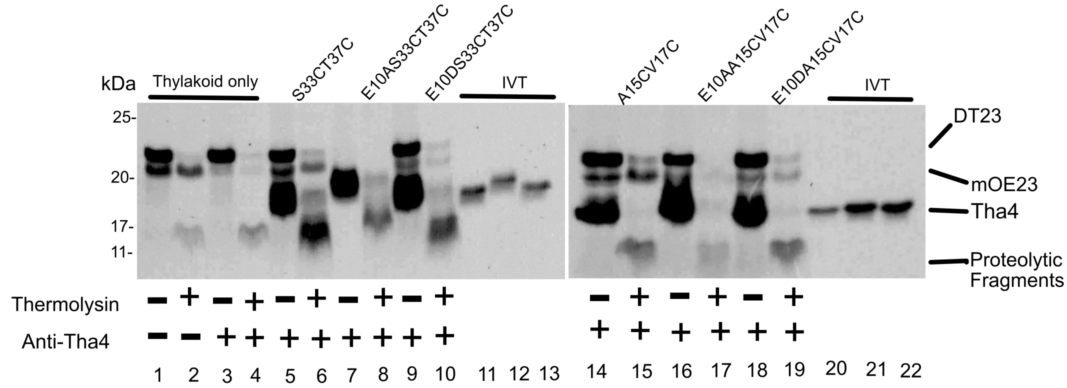

Figure S2: The Tha4 double cysteine variants S33CT37C and A15CV17C restore cpTat transport. Wild-type (WT) and double cys Tha4 variants in the E10A and E10D background were incubated with isolated thylakoids treated with α-Tha4 antibody to block the cpTat translocation. Thylakoid-only lanes indicate the antibody-treated thylakoids without the in vitro translated Tha4 variant. Transport was initiated by adding radiolabeled DT23 precursor and incubating thylakoids in light for 20 minutes. All samples were split and treated with either thermolysin protease (+) or buffer. Transport is indicated by the formation of protease-protected mature DT23. The successful transformation is demonstrated by mOE23 at approximately 23 kDa in the lanes that are treated with wild-type variants (lanes 2,6, and 15). DT23 precursor appears at around 25 kDa in non-thermolysin-treated wells (lanes 5,7,914,16, and 18). E10A variants did not show bands around ~23 kDa, indicating precursor transport. Complementation assays were carried out as described in the Materials and Methods section and analyzed by 12.5% SDS-PAGE under non-reducing conditions. Lanes 11,12,13, 19,20 and 22 show the translated products of the Tha4 variants, S33CT37C, E10AS33CT37C, E10DS33CT37C, A15CV17C, E10AA15CV17C, and E10DA15CV17C, respectively.

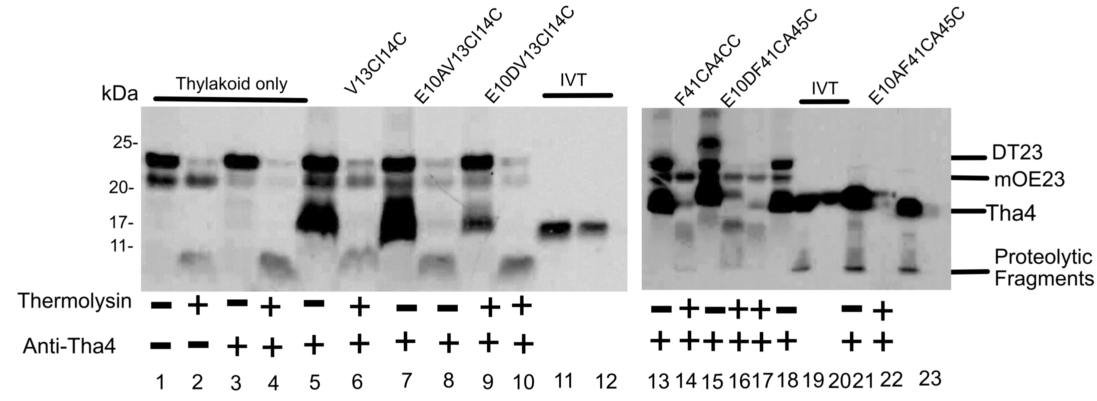

Figure S3: The Tha4 double cysteine variants V13CI14C and F41CA45C restore cpTat transport. Wild-type (WT) and double cysteine Tha4 variants from the E10A and E10D backgrounds were incubated with isolated thylakoids that had been treated with α-Tha4 antibody to inhibit cpTat translocation. The lanes containing only thylakoids demonstrate the antibody-treated thylakoids without any in vitro translated Tha4 variant. Transport commenced upon the addition of radiolabeled DT23 precursor, followed by a 20-minute incubation of the thylakoids in light. All samples were divided and treated with either thermolysin protease (+) or buffer. Transport is indicated by the emergence of protease-protected mature DT23. The successful transformation is shown by mOE23 at approximately 23 kDa. Typically, wild-type variants display bands around 23 kDa,

indicating mature DT23; the E10D variant also indicates mature DT23 in their complementation reactions (lanes 2, 6,8,14 and, 16). Complementation assays were conducted as detailed in the Materials and Methods section and analyzed using 12.5% SDS-PAGE under non-reducing conditions. Lanes 11,12,19,20, and 23 display the in vitro translated products (IVT) of the Tha4 variants: V13CI14C, E10AV13CI14C, F41CA45C, E10AF41CA45C, and E10DF41CA45C, respectively.

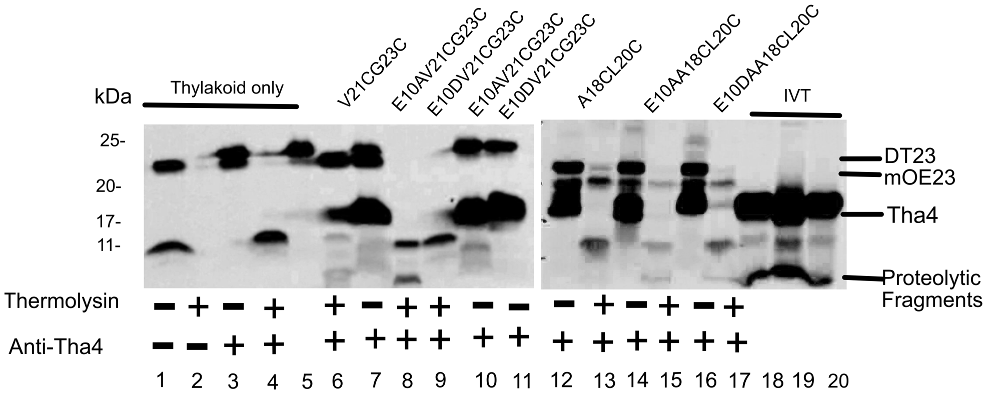

Figure S4: Tha4 double cysteine variants V21CG23C and A18CL20C restore cpTat transport. Wild-type (WT) and double cysteine Tha4 variants from the E10A and E10D backgrounds were incubated with isolated thylakoids that had been treated with α-Tha4 antibody to inhibit cpTat translocation. The lanes containing only thylakoids demonstrate the antibody-treated thylakoids without any in vitro translated Tha4 variant. Transport commenced upon the addition of radiolabeled DT23 precursor, followed by a 20-minute incubation of the thylakoids in light. All samples were divided and treated with either thermolysin protease (+) or buffer. Transport is indicated by the emergence of protease-protected mature DT23. The successful transformation is indicated by mOE23 at approximately 23 kDa (in lanes 2,6 and 13). Complementation assays were conducted as detailed in the Materials and Methods section and analyzed using 12.5% SDS-PAGE under non-reducing conditions. Lanes 5, 19, 20, and 22 display the in vitro translated (IVT) products of the Tha4 variants, DT23, A18CL20C, E10A A18CL20C, and E10D A18CL20C respectively.

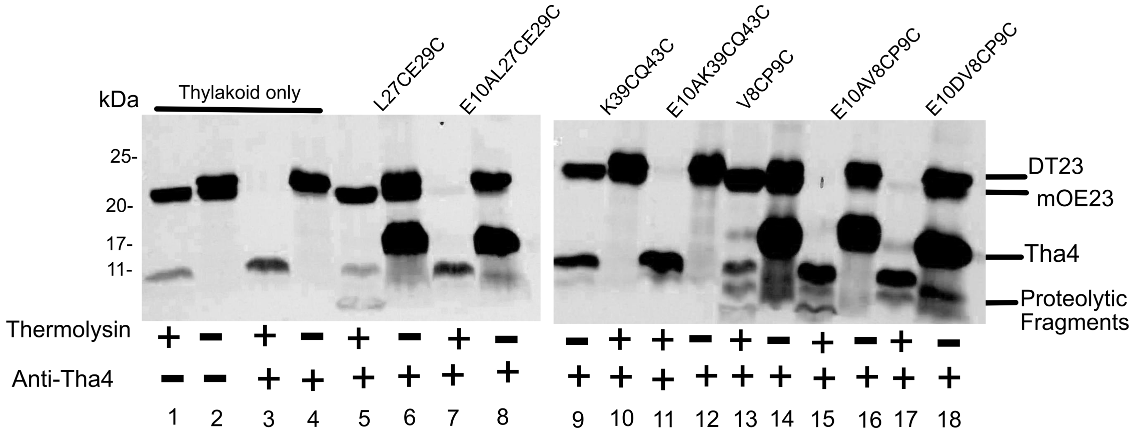

Figure S5: The Tha4 double cysteine variants L27CE29C, V8CP9C and K39CQ43C restore cpTat transport. Wild-type (WT) and double cysteine Tha4 variants from the E10A and E10D backgrounds were incubated with isolated thylakoids that had been treated with α-Tha4 antibody to inhibit cpTat translocation. The lanes containing only thylakoids demonstrate the antibody-treated thylakoids without any in vitro translated Tha4 variant. Transport commenced upon the addition

of radiolabeled DT23 precursor, followed by a 20-minute incubation of the thylakoids in light. All samples were divided and treated with either thermolysin protease (+) or buffer. Transport is indicated by the emergence of protease-protected mature DT23. The successful transformation is indicated by mOE23 at approximately 23 kDa (in lanes 2,5, 10 and 13). Complementation assays were conducted as detailed in the Materials and Methods section and analyzed using 12.5% SDS-PAGE under non-reducing conditions.

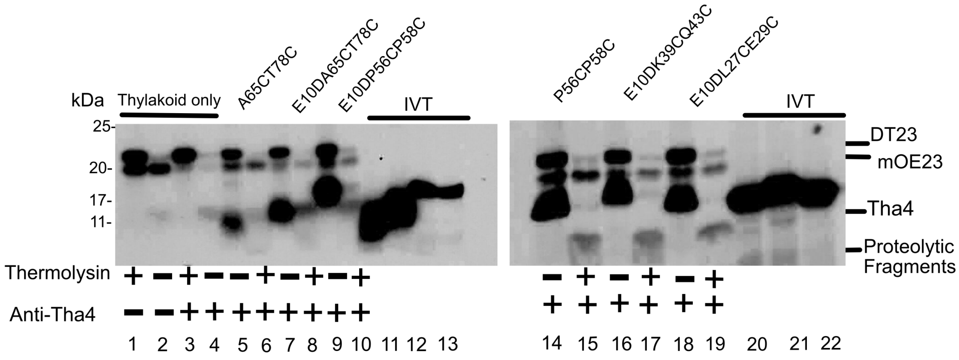

Figure S6: The Tha4 double cysteine variants A65CT78C, P56CP58C, E10D K39CQ43C and E10DL27CE29C restore cpTat transport. Wild-type (WT) and double cysteine Tha4 variants from E10D backgrounds were incubated with isolated thylakoids that had been treated with α-Tha4 antibody to inhibit cpTat translocation. The lanes containing only thylakoids demonstrate the antibody-treated thylakoids without any in vitro translated Tha4 variant. Transport commenced upon the addition of radiolabeled DT23 precursor, followed by a 20-minute incubation of the thylakoids in light. All samples were divided and treated with either thermolysin protease (+) or buffer. Transport is indicated by the emergence of protease-protected mature DT23. The successful transformation is indicated by mOE23 at approximately 23 kDa (in lanes 2,6,8,10, 15,17, and 19). Complementation assays were conducted as detailed in the Materials and Methods section and analyzed using 12.5% SDS-PAGE under non-reducing conditions. Lanes 11,12,13,20,21, and 22 display the in vitro translated A65CT78C, E10DA65CT78C, E10DP56CP58C, P56CP58C, E10D K39CQ43C, and E10DL27CE29C respectively.

Crosslinking Gel images.

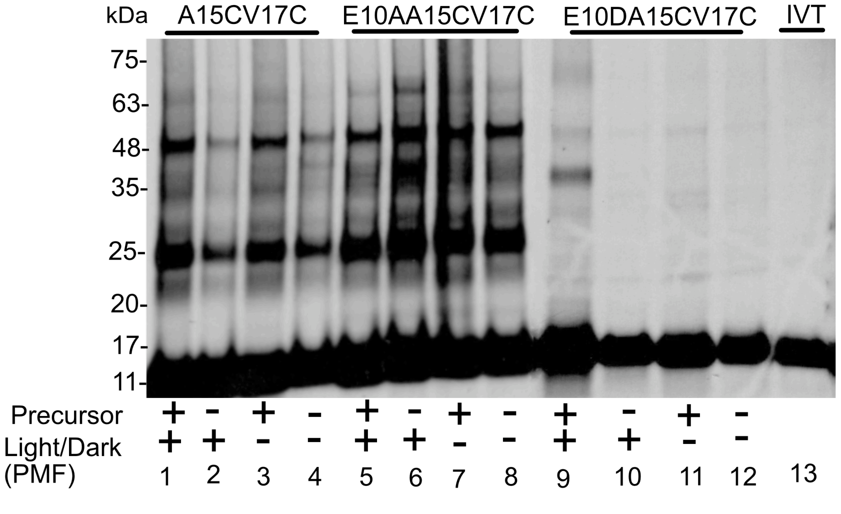

Figure S7: The formation of crosslinking in the A15CV17C relies on the existence of a precursor rather than on the proton motive force (PMF). The crosslinking reactions were conducted with and without the functional precursor tOE17, which has a size of approximately 17 kDa. These experiments were performed under both light and dark conditions to investigate oligomer formation in relation to PMF availability. All the crosslinking reaction mixtures were normalized to equal chlorophyll concentrations mentioned in the methods and materials section.

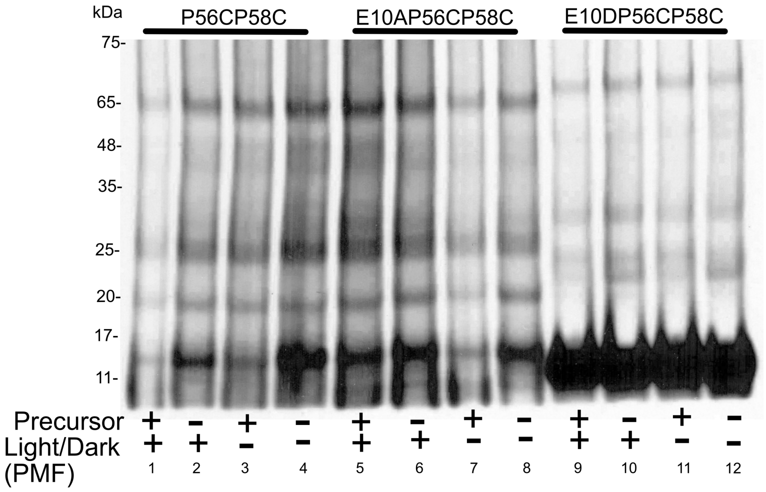

Figure S8: The formation of crosslinking in the P56CP58C in the C-tail of the Tha4. The crosslinking reactions were conducted with and without the functional precursor tOE17, which has a size of approximately 17 kDa. These experiments were performed under both light and dark conditions to investigate oligomer formation in relation to PMF availability. All the crosslinking reaction mixtures were normalized to equal chlorophyll concentrations mentioned in the methods and materials section.

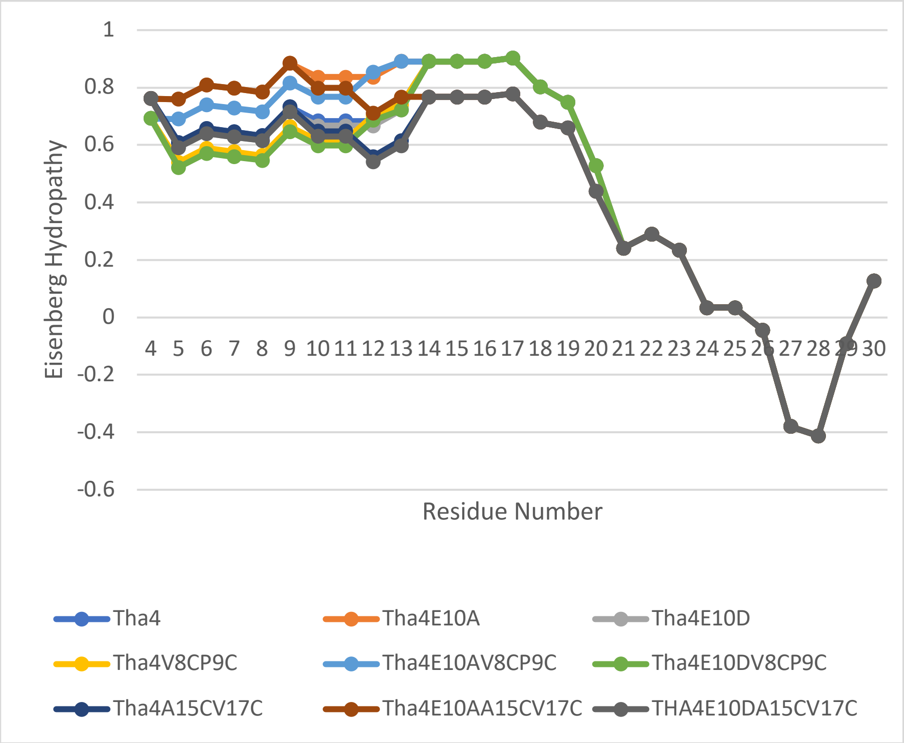

Figure S9: Eisenberg hydrophobicity values of residues 5- 30 Tha4 in cysteine-free and double-cysteine Tha4 E10/A/D variants. Comparison between Tha4 E10/A/D and Tha4 E10/A/D V8C P9C and A15CV17C residues indicated. The Eisenberg hydrophobicity values were determined using ExPASy ProtScale, with a residue window set to n = 9. This employed a linear weight variation model, assigning window edge residue weights at 10% relative to the central residue i (where i = 100%, i ± 1 = 78%, i ± 2 = 55%, i ± 3 = 33%, and i ± 4 = 10%).

Gel images of Tha4 crosslinking assays analyzed with Image-J plots

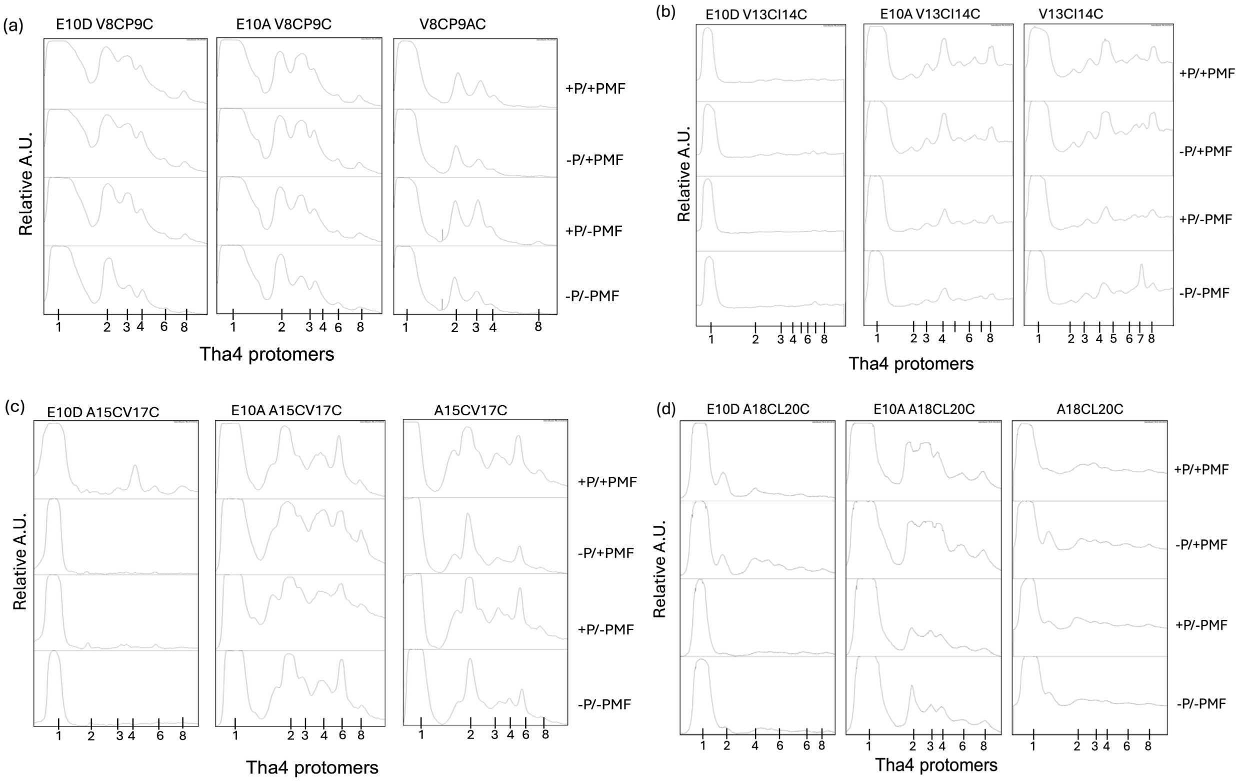

Figure S10: Crosslinking formation in the TMH region quantified using ImageJ. Each crosslinking gel image was analyzed using ImageJ, a software developed by the National Institutes of Health. Each band formed by crosslinks between the double cysteine residues in the presence and absence of the precursor, labeled P, and with or without PMF, was quantified by ImageJ and plotted. ImageJ plots for V8CP9C, V13CI14C, A15CV17C, and A18CL20C are shown in panels (a), (b), (c), and (d), respectively.

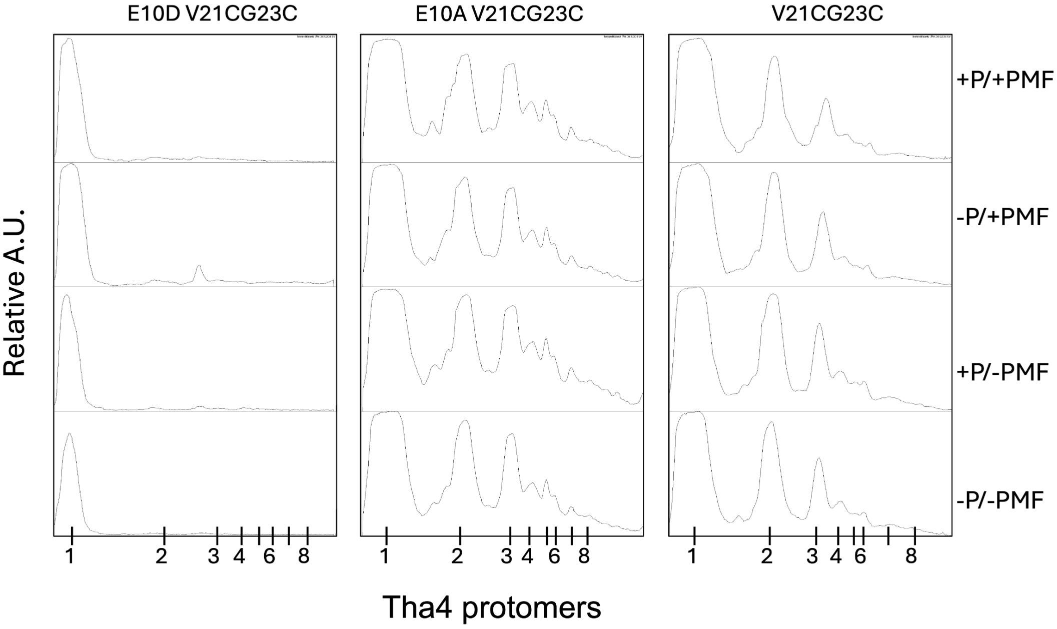

Figure S11: Crosslinking formation in the hinge region, V21CG23C quantified by using Image J. Each of the crosslinking gel images was analyzed with Image J, developed by the National Institutes of Health, to quantify each band in order to investigate the crosslinking formation of the double cys residues in the presence and absence of the precursor denoted by P and in the presence and absence of PMF (PMF).

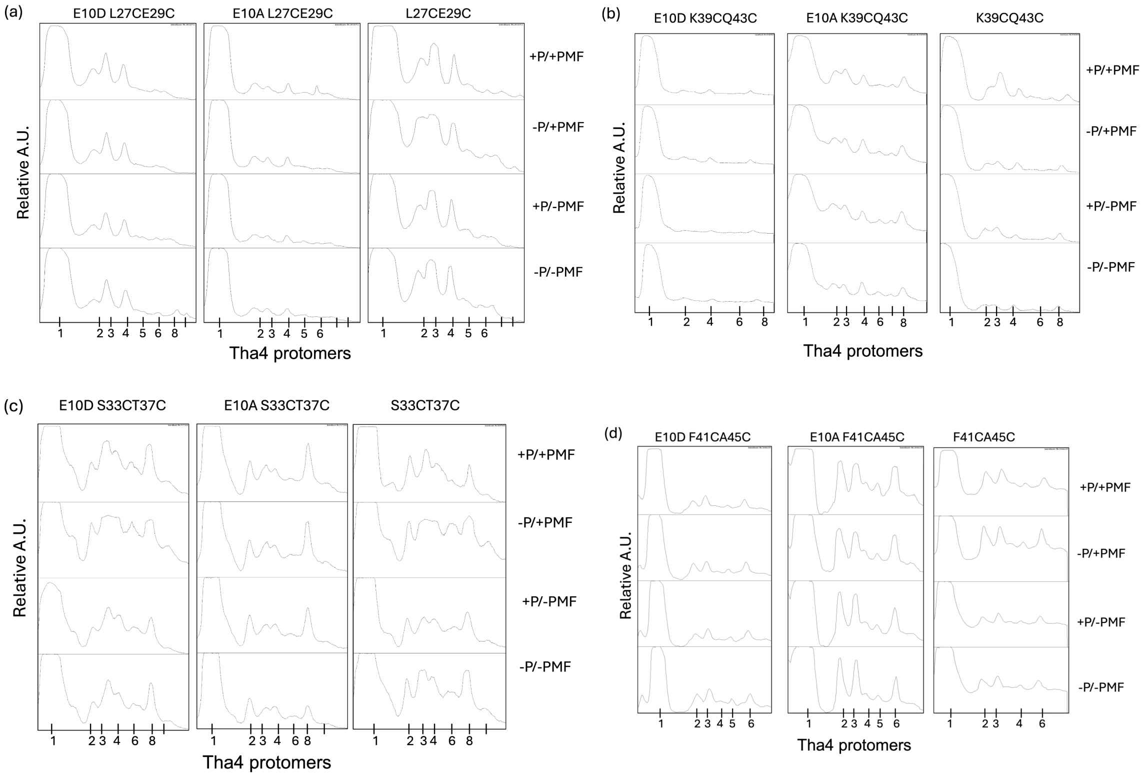

Figure S12: Crosslinking formation in the APH region quantified by using Image J. Each of the crosslinking gel images was analyzed with Image J, developed by the National Institutes of Health, to quantify each band in order to investigate the crosslinking formation of the double cys residues in the presence and absence of the precursor denoted by P and in the presence and absence of PMF. ImageJ plots for L26CG29C, S33CT37C, K39CQ43C, and F41CA45C are shown in panels (a), (b), (c), and (d), respectively.

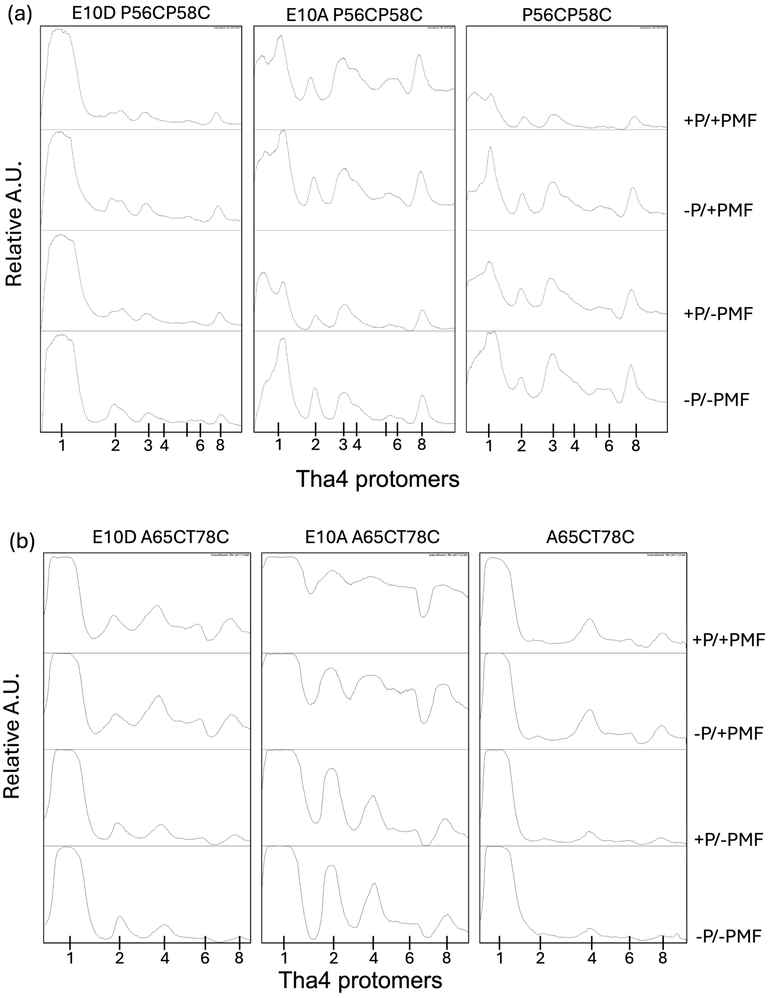

Figure S13: Crosslinking formation in the C tail region quantified by using Image J. Each of the crosslinking gel images was analyzed with Image J, developed by the National Institutes of Health, to quantify each band in order to investigate the crosslinking formation of the double cys residues in the presence and absence of the precursor denoted by P and in the presence and absence of PMF. P56CP58C and A65CT78Care shown in panels (a) and (b) respectively.
